# Imatinib Desensitization After a Type IV Hypersensitivity Reaction in a Gastrointestinal Stromal Tumor Patient—A Case Report

**DOI:** 10.1002/cnr2.70238

**Published:** 2025-06-11

**Authors:** Maud B. A. van der Kleij, Tristan V. M. Bruijn, Raween W. Kalicharan, Yannick S. Elshot, Maxime C. F. Pilon, Mark Oostdijk, Matthijs M. Tibben, Bastiaan Nuijen, Alwin D. R. Huitema, Thomas Rustemeyer, Neeltje Steeghs

**Affiliations:** ^1^ Department of Clinical Pharmacology, Division of Medical Oncology The Netherlands Cancer Institute, Antoni van Leeuwenhoek Amsterdam the Netherlands; ^2^ Department of Medical Oncology Erasmus MC Cancer Institute, Erasmus University Medical Center Rotterdam the Netherlands; ^3^ Department of Dermatology The Netherlands Cancer Institute, Antoni van Leeuwenhoek Amsterdam the Netherlands; ^4^ Department of Dermatology Amsterdam University Medical Center, University of Amsterdam Amsterdam the Netherlands; ^5^ Department of Pharmacy & Pharmacology The Netherlands Cancer Institute, Antoni van Leeuwenhoek Amsterdam the Netherlands; ^6^ Department of Medical Oncology The Netherlands Cancer Institute, Antoni van Leeuwenhoek Amsterdam the Netherlands; ^7^ Department of Clinical Pharmacy Utrecht University Medical Centre, Utrecht University Utrecht the Netherlands; ^8^ Department of Pharmacology Princess Máxima Centre for Pediatric Oncology Utrecht the Netherlands; ^9^ Department of Dermatology‐Allergology and Occupational Dermatology Amsterdam University Medical Centre Amsterdam the Netherlands; ^10^ Department of Medical Oncology Utrecht University Medical Centre, Utrecht University Utrecht the Netherlands

**Keywords:** case report, desensitization, gastrointestinal stromal tumor, imatinib, type IV hypersensitivity reaction

## Abstract

**Background:**

Imatinib treatment is approved for several indications, including chronic myeloid leukemia (CML) and gastrointestinal stromal tumors (GIST). Although adverse events are common, hypersensitivity reactions are not. Because there is a clear clinical benefit of imatinib treatment, re‐introduction of imatinib after a hypersensitivity reaction should be considered.

**Case:**

Here we present a case report of a 68‐year‐old patient with a GIST diagnosis who was re‐introduced to imatinib after a type IV hypersensitivity reaction via desensitization. A desensitization plan, a plan for formulation of low‐dose imatinib capsules, and the essential steps when considering desensitization are discussed.

**Conclusion:**

Our case of a patient with a type IV hypersensitivity reaction after starting imatinib treatment demonstrates that desensitization is a feasible option after serious cutaneous adverse events in specific cases, when done with good interdisciplinary collaboration and clinical management.

## 1 Introduction

Imatinib is a tyrosine kinase inhibitor that targets the breakpoint cluster region‐Abelson (BCR‐ABL), cKIT, and platelet‐derived growth factor receptor alpha (PDGFRα) gene. Imatinib is used to treat several malignancies, including Philadelphia chromosome‐positive chronic myeloid leukemia (CML) and KIT‐mutated gastrointestinal stromal tumors (GIST). For both indications, clinical efficacy has been demonstrated to be highly significant [1]. For example, for GIST—a rare type of mesenchymal neoplasms of the gastrointestinal tract—the introduction of imatinib improved median overall survival from 9 months with chemotherapy and radiation to 57 months with imatinib treatment [2]. As imatinib pharmacokinetic (PK) levels are correlated with more prolonged survival, achieving minimal plasma concentrations (*C*_min_) of => 1000 ng/mL (CML) and => 1100 ng/mL (GIST) is an additional treatment goal [3, 4]. As there is also a correlation between exposure and toxicity, it is critical to avoid excessively high exposure, although there is no well‐defined PK threshold for GIST. For CML, however, AE incidence has been associated with *C*_min_ of > 3000 ng/mL [4, 5]. Common adverse events from imatinib include nausea, vomiting, abdominal pain, diarrhea, fatigue, myalgia, muscle cramps, and cutaneous reactions [6]. Although adverse events are common, hypersensitivity reactions are not (< 0.01%) [6]. Hypersensitivity reactions come in different types, from IgE‐mediated immediate reactions (type I) to delayed‐type T‐cell‐mediated hypersensitivity reactions (type IV), including drug rash with eosinophilia and systemic symptoms (DRESS) [7]. Because of the clear clinical benefit of imatinib treatment, it is essential to keep it available as a treatment option even when there is a history of serious adverse events, such as hypersensitivity reactions. As these hypersensitivity reactions are uncommon, treatment plans when there is a wish to continue imatinib treatment although a hypersensitivity reaction occurred are primarily described in case reports [8, 9, 10, 11, 12, 13, 14, 15]. Accordingly, descriptions of the treatment plans for hypersensitivity reactions are valuable.

## 2 Case

**FIGURE 1:**
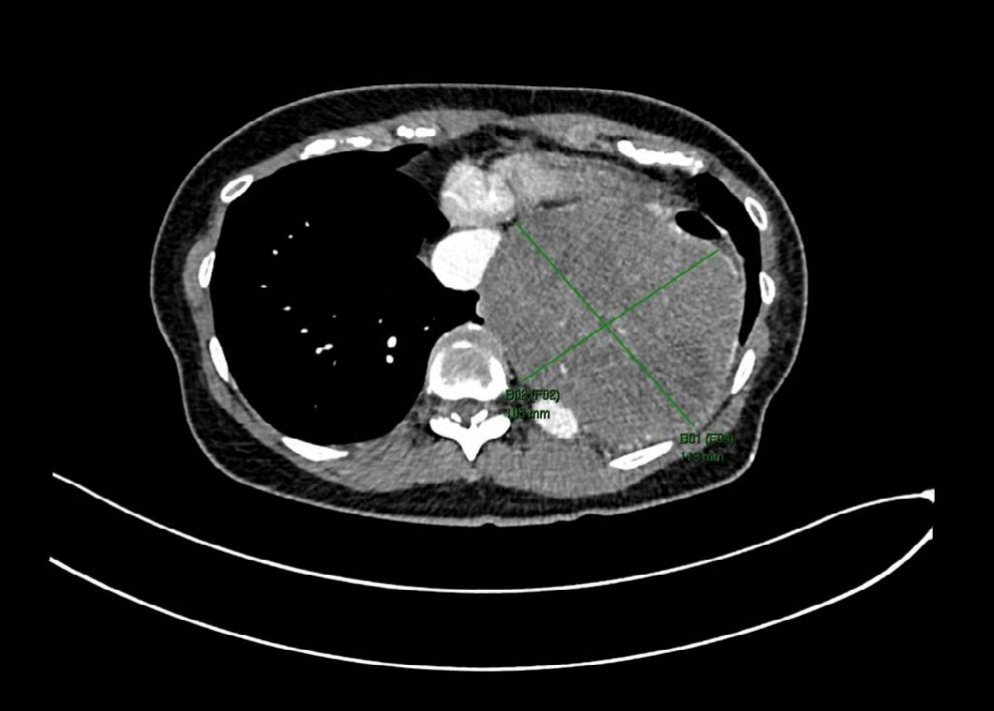
CT‐scan during initial diagnosis. Key image of the abdominal CT‐scan, transverse plane, made at initial diagnosis (2023). Marked in green is the gastrointestinal stromal tumor and the cross section measurements of 105*119 mm located in the lower esophagus.

A 68‐year‐old woman was diagnosed with a GIST of 10.5 × 11.9 × 11.3 cm originating from the lower esophagus (see Figure 1 for a key‐image of the computed tomography (CT)‐scan during initial diagnosis) in March 2023 at the Netherlands Cancer Institute, Amsterdam, The Netherlands. She was simultaneously diagnosed with stage 1 non‐small cell lung carcinoma, for which she was curatively treated with radiotherapy. Further medical history was not significant. GIST diagnosis was confirmed by pathology, with a low mitotic rate (≤ 5/5mm^2^), and primary KIT exon 11 and KIT exon 13 mutations. Imatinib 400 mg once daily (QD) was started as neoadjuvant therapy, at which the *C*_min_ was 944 ng/mL after 3 weeks of treatment (for a timeline of events, see Table 1).

**TABLE 1:** Timeline of events.

Date	Event
2023	
15–03	Start imatinib 400 mg QD
17–04	Start symptoms: drug‐induced hypersensitivity reaction
24–04	Start oral prednisone 0.5 mg/kg; Stop imatinib
28–04	Suspected type IV hypersensitivity reaction (DRESS); Hospitalization; Start oral prednisolone 1 mg/kg
08–05	No improvement of the skin reaction; start hydroxyzine and betamethasone cream
02–06 till 25–08	Clearance of symptoms. Prednisone and betamethasone cream were tapered
17–07	CT‐scan: Partial response
25–08	Prednisone stopped completely
25–09	Restart imatinib 100 mg QD
27–09	Recurrence of symptoms with dyspnea; type I hypersensitivity reaction considered; hospitalization; start oral prednisone 0.5 mg/kg; stop imatinib
28–09	Symptoms worsening; start oral prednisone 1 mg/kg and betamethasone cream
02–10 till 20–10	Symptoms improved; prednisone and betamethasone cream were tapered
20–10	Prednisone stopped completely
01–11	CT‐scan: Metastatic disease
06–11 till 09–11	Intradermal and epicutaneous allergy tests suggest type IV hypersensitivity reaction.
08–11	Start sunitinib
2024	
23–02	CT‐scan: Stable disease
22–04	Start imatinib desensitization: start imatinib 1 mg QD
29–04	Start imatinib 3 mg QD
06–05	Start imatinib 10 mg QD
11–05	Cutaneous symptoms (erythema and pruritus).
15–05	Start imatinib 30 mg QD; stop sunitinib because of oral mucositis.
21–05	CT‐scan: Stable disease
22–05	Worsening of cutaneous symptoms. Start betamethasone cream
23–05	After improvement of symptoms; start imatinib 50 mg QD
28–05	Start imatinib 100 mg QD
03–06	Start imatinib 300 mg QD
04–06	Reoccurrence of cutaneous symptoms; decrease to imatinib 100 mg QD
05–06	Stabilization of pruritus and improvement of erythema; start imatinib 200 mg QD
06–06	Improvement of symptoms; start imatinib 300 mg QD
09–06	Reoccurrence of cutaneous symptoms; decrease to imatinib 200 mg QD
11–06	Stop imatinib; restart sunitinib
22–08 and 25–11	CT‐scan: Stable disease.

Abbreviations: CT: computed tomography; QD: quaque die = once daily.

**FIGURE 2:**
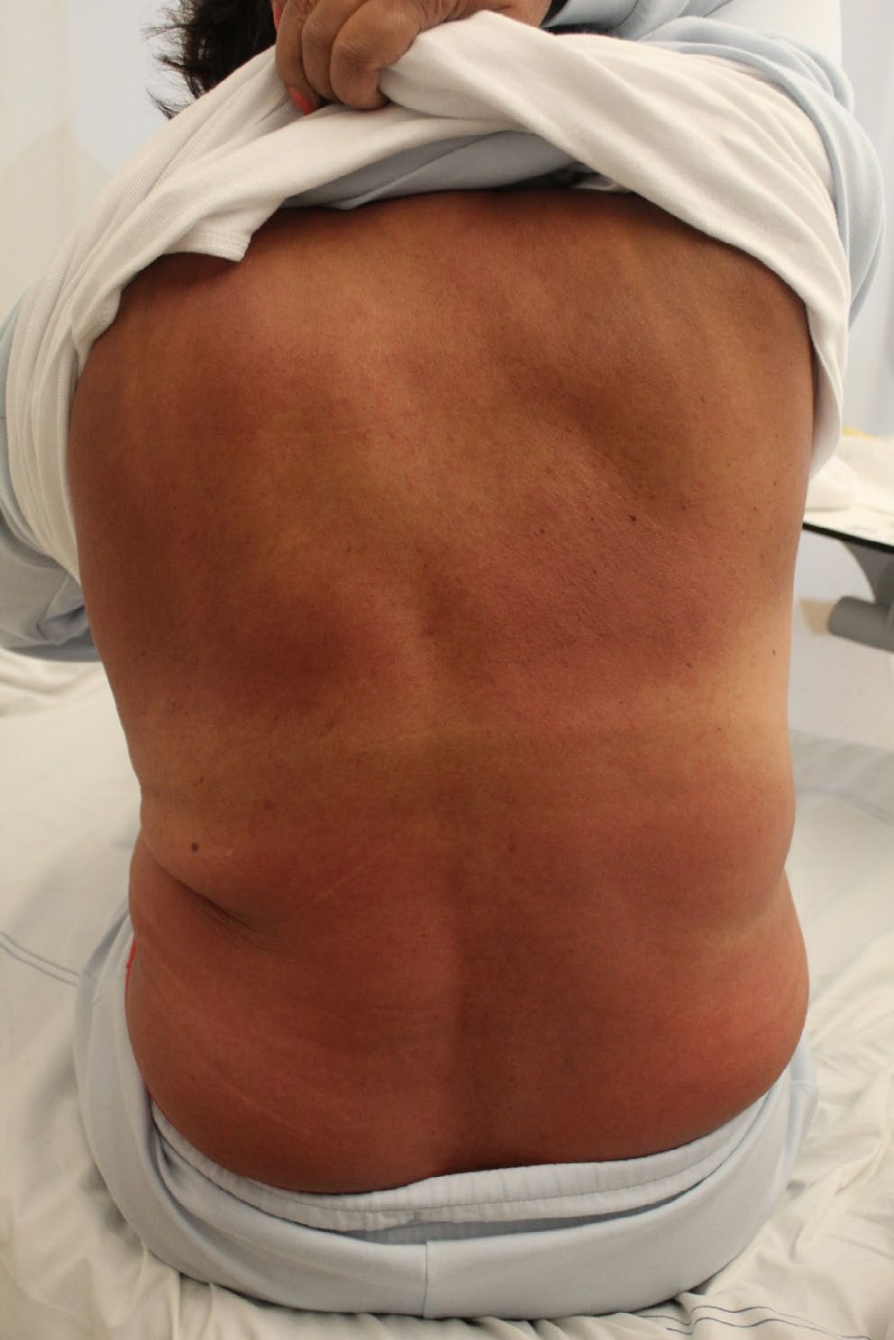
Photograph of erythema. Photograph of the patient's back at the time of first cutaneous adverse events after starting imatinib treatment consisting of a near confluent, diffuse, livid macular erythema.

**FIGURE 3:**
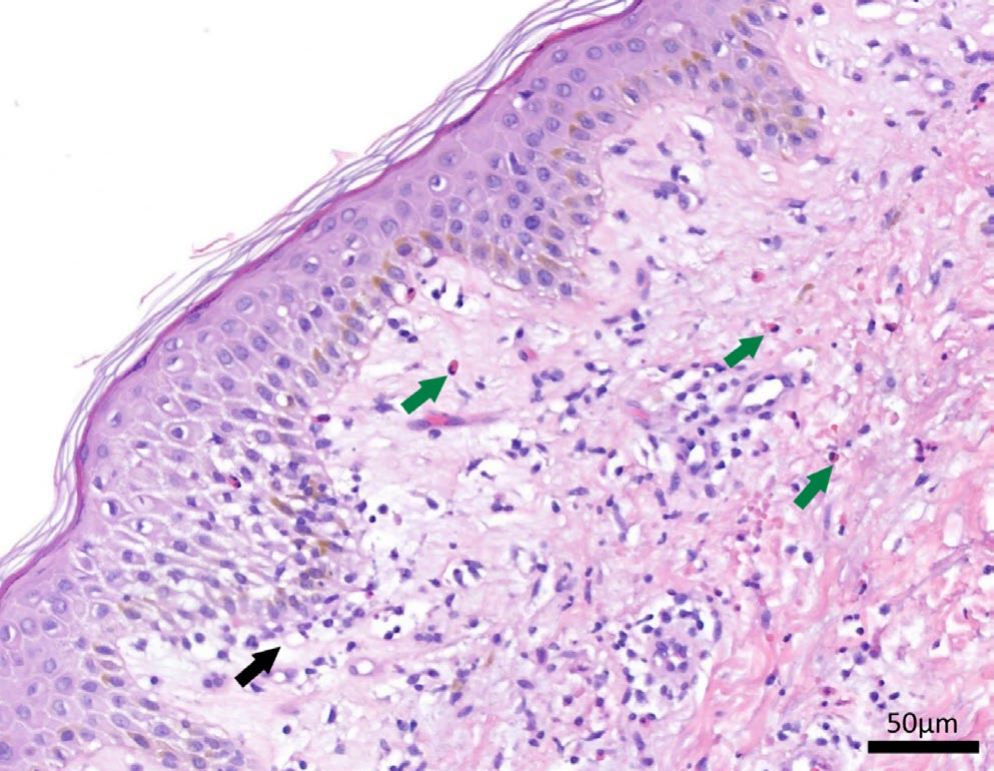
Histology of skin biopsy. Histology of skin biopsy showing a vacuolar interface dermatitis (black arrow) with mild spongiosis, subepidermal edema, and a perivascular infiltrate with multiple eosinophils (green arrows).

After 4 weeks of treatment, the patient reported progressive generalized confluent pruritic (maximum visual analogue scale [VAS]‐score of 10) macular erythema with desquamation of the upper extremities. The dermatology department diagnosed the patient with drug‐induced exfoliative dermatitis (affected body surface 70%) in addition to non‐palpable petechiae on the lower extremities (see Figure 2 for a picture of the erythema). A skin biopsy was performed before oral prednisolone 0.5 mg/kg QD was initiated for 7 days. Imatinib use was temporarily suspended. Four days later, the erythema progressed further, with increased desquamation and the development of systemic symptoms: general discomfort, facial and peripheral edema, enlarged lymph nodes, and fever. Laboratory test results revealed leukocytosis, neutrophilia, and eosinophilia. Urinary sediments showed mild proteinuria and leukocyturia. Liver function tests were normal (See Table S1 for laboratory results). EBV, CMV, and HSV type 1/2 serologies were IgG‐positive and IgM‐negative, ruling out viral infection or reactivation. A type IV hypersensitivity diagnosis—and more specifically, DRESS—was considered. The DRESS Registry of Severe Cutaneous Adverse Reactions score (RegiSCAR score) was 3–4 (possible to probable diagnosis). Because of the severity of symptoms, the patient was admitted to the clinic. Prednisone dose was increased to 1 mg/kg QD orally, and prophylactic pantoprazole and trimethoprim/sulfamethoxazole were started. Skin biopsy revealed vacuolar interface dermatitis with mild spongiosis, subepidermal edema, and a superficial perivascular and interstitial infiltrate with limited eosinophilia, consistent with a drug‐induced hypersensitivity reaction. See Figure 3 for the histological image. Systemic symptoms improved after 2 days. After 6 weeks of prednisolone treatment, the cutaneous symptoms improved and prednisolone was slowly tapered.

CT‐scan showed a partial tumor response after a combined 1 month of imatinib treatment and 1 month of interruption of imatinib treatment. Five months after the initial interruption of imatinib treatment and 1 month after prednisone was completely tapered, imatinib was re‐introduced at a lowered dose of 100 mg QD. After 2 days of treatment, the pruritic confluent macular erythema recurred, coinciding with dyspnea and chills. Because of the rapid onset of symptoms, including dyspnea, a diagnosis of a type I hypersensitivity reaction was now also taken into consideration. Imatinib was stopped again. Prednisolone 0.5 mg/kg QD was restarted, and after progression of the cutaneous symptoms, it was increased to 1 mg/kg QD. After 5 days of treatment, the cutaneous symptoms improved, and prednisolone was tapered again.

The patient was referred to the Dermatology‐Allergology and Occupational Dermatology Department to discern between a delayed type IV or an immediate type I hypersensitivity reaction. Due to the variety in onset of symptoms and the type of symptoms, differentiating between these was difficult. Epicutaneous allergy tests (used to diagnose delayed hypersensitivity reactions) were considered difficult to evaluate due to poor skin penetration of imatinib and indeed remained negative. Hence, intradermal tests (mainly used to diagnose immediate hypersensitivity reactions, but also allow for testing of delayed reactions) were found to be positive. In conclusion, the diagnosis of a type IV hypersensitivity reaction to imatinib was made based on medical history, histopathological and laboratory tests and confirmed by allergological investigation.

One and a half months after the second interruption of imatinib treatment, a CT scan showed metastatic disease. Treatment with sunitinib was initiated.

**FIGURE 4:**
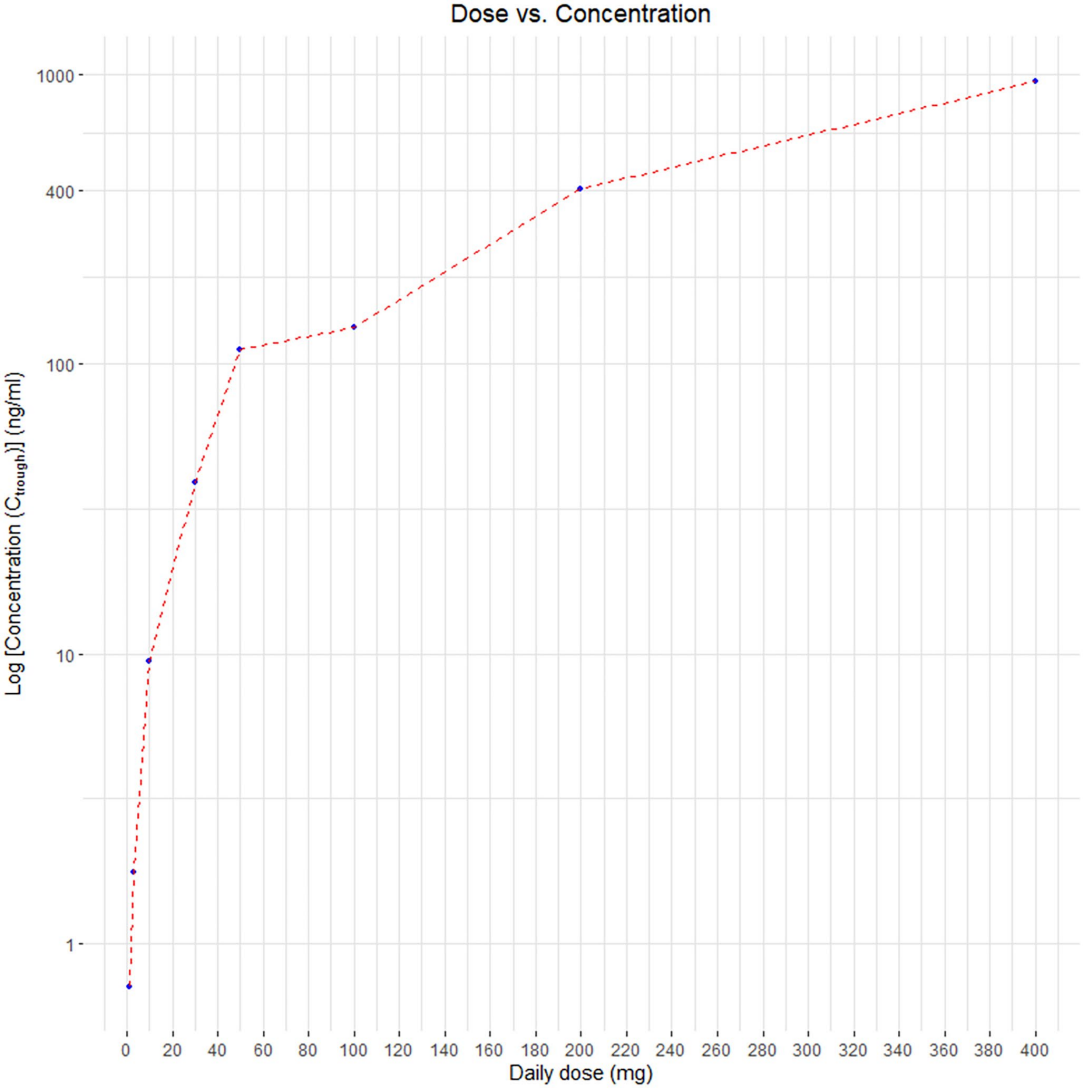
The daily imatinib dose in relation to the log concentration. This graph shows the imatinib dose in relation to the log imatinib plasma concentration. Imatinib dose varied from 1 to 400 mg QD, although this was not chronological. The concentration measured on 200 mg QD might be influenced by the switch from 300 to 200 mg QD 2 days before PK measurement, as the 300 mg is expected to give higher PK levels and imatinib half‐life is 18 h; the influence of this higher dose on exposure might not have cleared. C_trough_: minimal plasma concentration; QD: quaque die = once daily.

Desensitization to imatinib was planned in consultation with an allergologist/dermatologist. The pharmacy was consulted to manufacture low‐dose imatinib capsules, which took approximately 1 month. This desensitization was combined with sunitinib treatment, so that tumor control could be retained with the start of very low imatinib doses. The plan was to increase imatinib weekly with an approximately three‐fold increase (1 mg QD–3 mg QD–10 mg QD–30 mg QD–100 mg QD–300 mg QD–400 mg QD) after clinical assessment, PK sampling (See Figure 4 and Table S2 for PK results), and laboratory testing (See Table 2 for laboratory results) for indications of systemic symptoms for DRESS diagnosis. The patient stayed in the hospital for 3 days after every increase to monitor hemodynamic parameters and possible delayed hypersensitivity symptoms. While the first two increments went without problems, the patient developed pruritus and erythema at the end of the 10 mg QD week. No additional laboratory abnormalities were observed, and the symptoms resolved spontaneously within 2 days. Sunitinib treatment was temporarily discontinued due to toxicity (oral mucositis). Imatinib was increased to 30 mg QD, but cutaneous symptoms recurred in exacerbated form. The symptoms improved after the application of betamethasone cream. A biopsy confirmed a drug‐induced hypersensitivity reaction. Imatinib was increased with an extra safety step (50 mg QD–100 mg QD–300 mg QD). Within 1 day of increasing the dose to 300 mg QD, the cutaneous symptoms recurred, and imatinib was lowered to the previously tolerated 100 mg QD dose. As the cutaneous symptoms did not progress, the dose was increased to 200 mg QD and 2 days later to 300 mg QD. However, the cutaneous symptoms progressed slightly and pruritus reappeared; therefore, the dose had to be decreased to 200 mg QD, which was well tolerated. Imatinib desensitization up to 200 mg QD was considered successful. Imatinib *C*_min_ at the 200 mg dose was 402 ng/mL. As sunitinib was still proven to be clinically active (CT scan showed stable disease), it was decided to restart and continue sunitinib until disease progression. Subsequently, in case of disease progression, we plan to restart imatinib with a short inclined schedule with weekly dose increments (25 mg QD–50 mg QD–100 mg QD–200 mg QD) and, if feasible, even higher.

**TABLE 2:** Steps for when desensitization is considered.

Step 1	Outweigh the possible treatment effect and the risk for re‐introduction of the drug.
Step 2	Consult the allergologist about the possibilities and safety of desensitization.
Step 3	Discuss with the pharmacist about the possibilities of producing low doses of the drug.
Step 4	Make a clear desensitization plan and discuss thoroughly with your team.
Step 5	Start desensitization while the patient is hospitalized, while hospital staff is thoroughly informed about possible drug reactions.
Step 6	Always discuss the steps with your patient.

## 3 Discussion

Cutaneous reactions are one of the most common side effects of imatinib, as already described in a registration study for metastatic GIST and CML, in which 30.6% and 33.9% of patients developed rash/dermatitis, respectively. These were mainly grade 1–2, and grade 3–4 in 2.7% and 2.0%, respectively, of the patients [16, 17]. Later reports, including GIST patients treated in the adjuvant setting, described a similar dermatological safety profile [18, 19, 20]. The clinical presentation of cutaneous reactions ranges from maculopapular eruptions to lichenoid and psoriasiform dermatitis [21]. Cutaneous reactions to imatinib are dose dependent, and most often present in doses above 600 mg QD [22]. Although the mechanism causing the cutaneous reaction has not been elucidated yet, it is assumed that these reactions are in most cases caused by on‐target drug effects, and not triggered by an immunogenic response. For example, as c‐KIT is involved in melanocyte physiology, this likely explains the pigment disorder which can be seen during imatinib treatment [21, 23, 24]. On the other hand, maculopapular eruptions can likely be explained by a direct pharmacological effect, or in rare cases by an allergic mechanism [24, 25]. Hypersensitivity reactions most commonly manifest as cutaneous reactions, presenting without systemic symptoms such as fever and involvement of other organs [26]. Serious cutaneous adverse events, including more severe hypersensitivity reactions with systemic symptoms, have also been previously described in case reports [9, 10, 11, 12, 13, 14, 21, 27, 28, 29].

The types of hypersensitivity reactions are commonly distinguished by the onset of symptoms and the type of immune reaction. In our case, differentiating between type I and type IV was challenging because of the course of the cutaneous reactions. After starting imatinib, the patient experienced a hypersensitivity reaction after 4 weeks of treatment. Because of the delayed onset and type of symptoms, we suspected a type IV hypersensitivity reaction and potentially DRESS. DRESS is a potentially life‐threatening drug hypersensitivity reaction characterized by a morbilliform to polymorphic exanthema with fever, hematologic, and visceral organ involvement [30]. Because of the diversity in presentation, it is associated with diagnostic and management challenges for clinicians [30, 31]. Its incidence varies depending on the causative drug used. As the estimated mortality rate of DRESS ranges between 3.8% and 10%, early diagnosis, suspension of the drug culprit, and initiation of corticosteroid treatment are crucial [32].

In our patient, the suspicion of DRESS occurred during the generally present latency period of 2–6 weeks; DRESS was suspected based on clinical symptoms, and the diagnosis was supported by laboratory results and skin biopsy [32]. The DRESS RegiSCAR score suggested a probable case. Because both DRESS and imatinib are associated with the development of periorbital edema, the score could have been reduced to a possible outcome. Although DRESS is usually a contraindication for continuing therapy, in the case of our patient, there was no other neo‐adjuvant treatment available, and resection was not yet possible because of the tumor size and location. As a result, combined with the uncertainty of the DRESS diagnosis, we decided to reintroduce imatinib.

When imatinib was re‐introduced, generalized erythema recurred within 2 days. Although trigger re‐exposure can reduce the latency period, the combination with dyspnea in our patient could also indicate a potentially lethal anaphylactic hypersensitivity reaction. Intradermal tests with delayed readings were positive, suggesting a delayed hypersensitivity reaction. Noteworthy are the falsely negative epicutaneous allergy tests likely due to poor percutaneous penetration of imatinib.

When the patient progressed into the metastatic setting, sunitinib became accessible as an alternative treatment and was started. As disease progression on sunitinib would be unavoidable, and with the efficacy of imatinib during her first weeks of treatment in mind, we decided that desensitization for imatinib could result in clinical benefit for the patient, and starting desensitization was in the patient's best interest, with which the patient agreed. Our deliberations on the diagnosis of the type of hypersensitivity reaction made us additionally careful in our treatment plan, as a variety of reactions could possibly develop after re‐start of imatinib. During desensitization, we became more confident of a diagnosis of a type IV hypersensitivity reaction, as much more rapid onset of symptoms was expected with a type I hypersensitivity reaction than after 3 weeks of slow introduction of imatinib, or even after 1 week of 10 mg QD doses.

A desensitization plan differs between drugs and depends on the type of reaction, the type of drug, the dosing interval, the route of administration, and the availability of drug doses [33]. The plan should include the preferable planning, with dose adjustments, necessary laboratory tests and clinical assessments, and an emergency plan. Our plan was made based on the scarce imatinib desensitization cases found in the literature, expertise from an allergologist, and the availability of imatinib doses [6, 8, 9, 10, 11, 12, 13, 14, 15]. Execution of the plan was influenced by the clinical symptoms and wishes of the patient and altered when needed. In treating these patients, very close clinical follow‐up and multidisciplinary consultations are mandatory.

Imatinib is commercially available in 100 and 400 mg tablets. In this case, based on the literature [8], 1 and 10 mg oral formulations were needed, which are outside standard therapeutic practices and are unavailable commercially. Due to the unavailability of imatinib raw material within the required timeframe, the pharmacy compounded these doses by finely grinding 100 mg tablets and mixing them with microcrystalline cellulose. Capsules were prepared with 1 mg (*n* = 60) and 10 mg (*n* = 60) doses, achieving capsule weight deviations of 0.4% and 0.9% (within ±3% limits), and RSDs of 0.38% and 0.85% (within < 4% limits). As it is crucial to maintain both speed and flexibility during desensitization and since desensitization protocols often deviate from the initial plan, the pharmacy should be closely involved.

Although imatinib plasma concentrations were below the 1100 ng/mL target for GIST, and will most likely stay below 1100 ng/mL on the now tolerated lower dose, continuation of imatinib is still expected to be beneficial, compared to the standard third‐line GIST treatment regorafenib (median Time to Progression for patients with imatinib *C*_min_ < 1100 ng/mL: 11.3 months, compared to median Progression Free Survival for patients with third line regorafenib treatment of 4.8 months) [3, 34]. Based on future PK results, we will try to adjust the dose for the patient. As sunitinib was tolerated well with stable disease on CT, it was decided to first continue sunitinib treatment until progression.

To conclude, in specific cases, especially in a metastatic patient with no other treatment options, the possible treatment effect can outweigh the risk of re‐introduction of the treatment after a serious adverse event. However, this should be done in a controlled environment, in small steps, and in close contact with the allergologist (see Table 2 for the described steps when desensitization is considered). As also shown in our case, good interdisciplinary collaboration and clinical management allowed for successful personalized treatment after serious cutaneous adverse reactions.

## Author Contributions

Conceptualization: Maud B.A. van der Kleij, Thomas Rustemeyer, N.S. Formal analysis: Maud B.A. van der Kleij, Investigation: Maud B.A. van der Kleij, T.V.M.B., R.W.K., Y.S.E., M.C.F.P., M.O., M.M.T., T.R., N.S. Methodology: Maud B.A. van der Kleij, R.W.K., M.M.T., Thomas Rustemeyer, N.S. Resources: T.V.M.B., R.W.K., Y.S.E., M.M.T., B.N., A.D.R.H., T.R., N.S. Writing – original draft: Maud B.A. van der Kleij, T.V.M.B., R.W.K. Writing – review and editing: Maud B.A. van der Kleij, T.V.M.B., R.W.K., Y.S.E., M.C.F.P., M.O., M.M.T., B.N., A.D.R.H., T.R., N.S. Supervision: Y.S.E., B.N., A.D.R.H., N.S. Validation: B.N., A.D.R.H., Thomas Rustemeyer, N.S. Visualization: Maud B.A. van der Kleij All authors declare to be accountable for all aspects of the presented work and approved the final version.

## Ethics Statement

This study follows the CARE guidelines for case reports.

## Consent

The patient provided written consent to publish this case report and accompanying clinical images.

## Conflicts of Interest

Y.S.E. reports being a speaker for AbbVie, Bristol‐Myers Squibb, MSD, Novartis, Johnson & Johnson; all outside the submitted work. T.R. is on advisory boards and/or speaker for Leo Pharma, L'Oréal/La Roche Posay, Sanofi, UCB, MSD, Abbvie, Novartis, and LeoPharma, all outside the submitted work. T.R. reports research funding from LeoPharma, SmartPractice, and Vifor, all outside the submitted work. N.S. reports research grants paid to the institute from Abbvie, Actuate Therapeutics, Amgen, Array, Ascendis Pharma, AstraZeneca, Bayer, Blueprint Medicines, Boehringer Ingelheim, BridgeBio, Bristol‐Myers Squibb, Cantargia, CellCentric, Cogent Biosciences, Cresecendo Biologics, Cytovation, Deciphera, Dragonfly, Eli Lilly, Exelixis, Genentech, GlaxoSmithKline, IDRx, Immunocore, Incyte, InteRNA, Janssen, Kinnate Biopharma, Kling Biotherapeutics, Luszana, Merck, Merck Sharp & Dohme, Merus, Molecular Partners, Navire Pharma, Novartis, Numab Therapeutics, Pfizer, Relay Pharmaceuticals, Revolution Medicin, Roche, Sanofi, Seattle Genetics, Taiho, Takeda; all outside the submitted work. N.S. provided consultation or attended advisory boards for Boehringer Ingelheim, Cogent Biosciences, Ellipses Pharma, Incyte, Luszana; all outside the submitted work. The other authors declare no conflicts of interest.

## Supporting information


Table S1.

Table S2.

## Data Availability

The authors have nothing to report.
