# Supplementary material for: Imatinib Desensitization After a Type IV Hypersensitivity Reaction in a Gastrointestinal Stromal Tumor Patient—A Case Report
Source: Cancer Rep (Hoboken). 2025 Jun 11;8(6):e70238. doi: 10.1002/cnr2.70238 (PMC12153969; doi:10.1002/cnr2.70238)
Supplement: Supplementary file 1 — Table S1. [file CNR2-8-e70238-s002.docx]

**Supplementary table 1: Laboratory results**

| Laboratory / Date | Normal range | 24-04-23 | 28-04-23 | 30-04-23 | 22-04-24 | 29-04-24 | 06-05-24 | 14-05-24 | 21-05-24 | 23-05-24 | 28-05-24 | 03-06-24 | 11-06-24 |
| --- | --- | --- | --- | --- | --- | --- | --- | --- | --- | --- | --- | --- | --- |
| Imatinib daily dose (mg) |  | **400** | **0** | **0** | **0** | **1** | **3** | **30** | **30** | **30** | **50** | **100** | **200** |
| BSE | 0-20 mm/h | x | x | 55 | 55 | 30 | 34 | x | 23 | x | x | 30 |  |
| Hemoglobin | 7.5-10 mmol/L | x | 7.6 | 6.9 | 8.1 | 8.6 | 8.4 | 8.4 | 8.2 | 8.4 | 8.1 | 8.3 | 7.7 |
| Hematocrit | 0.35-0.45 | x | 0.37 | 0.35 | 0.39 | 0.41 | 0.41 | 0.40 | 0.40 | 0.41 | 0.39 | 0.41 | 0.38 |
| Erythrocytes | 4.0-5.0 x 10^12^/L | x | 4.1 | 3.7 | 3.9 | 4.1 | 4.1 | 4.0 | 4.0 | 4.0 | 3.9 | 4.0 | 3.8 |
| MCV | 80-100 fl | x | 91 | 94 | 99 | 100 | 101 | 101 | 101 | 101 | 100 | 101 | 100 |
| Leukocytes | 4.0-10.5 x 10^9^/L | x | 11.9 | 13.9 | 4.5 | 4.0 | 3.8 | 3.8 | 4.8 | 4.7 | 4.4 | 5.7 | 7.6 |
| Neutrophil granulocytes | 1.5-7.5 x 10^9^/L |  | 9.1 | 5.2 | 2.2 | 2.0 | 1.9 | 1.6 | 2.1 | 1.8 | 2.0 | 2.7 | 4.6 |
| Thrombocytes | 150-400 x 10^9^/L | x | 216 | 212 | 177 | 186 | 183 | 156 | 166 | 173 | 150 | 170 | 174 |
| Lymphocytes | 1.0-4.0 x 10^9^/L | x | 1.0 | 2.4 | 1.6 | 1.5 | 1.4 | 1.2 | 1.4 | 1.6 | 1.0 | 1.3 | x |
| Eosinophils | <0.5 x 10^9^/L | x | 1.6 | 5.0 | 0.2 | 0.2 | 0.6 | 0.6 | 0.9 | 0.8 | 0.8 | 1.1 | x |
| Total bilirubin | < 16 µmol/L | 9 | 6 | 7 | 7 | 5 | 5 | 6 | 4 | 5 | 5 | 3 | 4 |
| Direct bilirubin | < 4 µmol/L | x | 4 | 4 | - | 3 | 3 | 3 | 3 | 3 | 3 | 2 | - |
| Alkaline phosphatase | < 98 U/L | 77 | 66 | 62 | 61 | 70 | 64 | 56 | 71 | 60 | 55 | 66 | 62 |
| Aspartate aminotransferase | < 31 U/L | 25 | 20 | 19 | 30 | 27 | 29 | 26 | 25 | 25 | 24 | 31 | 30 |
| Alanine aminotransferase | < 34 U/L | 27 | 25 | 26 | 36 | 34 | 35 | 35 | 32 | 34 | 34 | 39 | 45 |
| Gamma-glutamyl transferase | < 38 U/L | 31 | 30 | 34 | 57 | 48 | 45 | 34 | 31 | 34 | 30 | 31 | 33 |
| Lactate dehydrogenase | < 247 U/L | x | 228 | 249 | 186 | 207 | 208 | 208 | 225 | 205 | 207 | 237 | 275 |
| C-reactive protein | < 8 mg/L | x | 11 | 5 | 2 | 2 | 3 | 3 | 2 | 2 | 2 | 2 | x |
| Creatinine | 40-95 µmol/L | 79 | 61 | 65 | 76 | 64 | 69 | 72 | 65 | 63 | 65 | 66 | 76 |
| EGFR (ckd-epi) | > 60 ml/min/1.73m^2^ | 67 | 90 | 84 | 69 | 84 | 78 | 74 | 84 | 86 | 84 | 82 | 69 |
| Sodium | 135-145 µmol/L | x | 139 | 143 | 139 | 141 | 142 | 139 | 140 | 141 | 141 | 141 | 140 |
| Potassium | 3.5-5.0 µmol/L | x | 4.1 | 3.6 | 4.1 | 3.7 | 3.7 | 3.8 | 3.7 | 3.8 | 3.7 | 3.8 | 3.8 |
| Phosphate | 0.80-1.50 µmol/L | x | 1.10 | 1.00 | 1.34 | 1.30 | 1.19 | x | 1.21 | 1.25 | x | 1.22 | 1.29 |
| Magnesium | 0.70-1.00 µmol/L | x | 0.91 | 0.96 | 0.94 | 0.93 | 0.88 | 0.89 | 0.86 | 0.95 | 0.88 | 0.90 | 0.92 |
| Glucose | 3.5-7.8 µmol/L | x | 10.1 | 5.0 | 4.4 | 6.0 | 4.3 | x | 6.2 | x | x | 3.3 | 3.8 |
| Total protein | 60-80 g/l | x | 73 | 77 | x | 82 | 80 | 77 | 77 | x | 76 | 78 | x |
| Albumin | 35-50 g/l | x | 42 | 43 | 49 | 50 | 48 | 47 | 47 | 48 | 46 | 45 | 47 |
| Calcium | 2.20-2.60 µmol/L | x | 2.26 | 2.42 | 2.39 | 2.39 | 2.41 | x | 2.37 | x |  | 2.39 | 2.29 |
| Creatine kinase | <145 U/l | x | x | x | 159 | 117 | 140 | 119 | 130 | 106 | 131 | 111 | x |
| CKMB-m | <4.9 µg/L | x | x | x | 2.4 | 2.0 | 2.6 | 1.9 | 2.2 | 1.7 | 2.1 | 2.3 | x |
| Total amylase | <107 U/L | x | x | x | 137 | 170 | 199 | 118 | 154 | 168 | 198 | 192 | x |
| Lipase | 15-65 U/l | x | 32 | x | 18 | 22 | 21 | 18 | 25 | 24 | 21 | 26 | 29 |
| Free thyroxine | 10-25 pmol/l | x | 18 | x | 16 | 14 | 15 | 16 | 298 | 14 | x | 16 | 15 |
| Thyroid-stimulating hormone | 0.500-4.20 mlU/l | x | 0.319 | x | 0.91 | 2.39 | 1.92 | 1.68 | 2.04 | 2.02 | x | 1.83 | 1.74 |
| Urine sample  leukocytes  protein | Normal | ++  + | x |  | + | + | + | + | + | x | x | Normal | x |
