# Supplementary material for: Imatinib Desensitization After a Type IV Hypersensitivity Reaction in a Gastrointestinal Stromal Tumor Patient—A Case Report
Source: Cancer Rep (Hoboken). 2025 Jun 11;8(6):e70238. doi: 10.1002/cnr2.70238 (PMC12153969; doi:10.1002/cnr2.70238)
Supplement: Supplementary file 2 — Table S2. [file CNR2-8-e70238-s001.docx]

**Supplementary table 2: Pharmacokinetic results**

| **Date** | **Imatinib dose during PK sample** (mg QD) | **(Calculated) trough level** (ng/mL) |
| --- | --- | --- |
| **2023** | | |
| 05-04 | 400 | 944* |
| **2024** | | |
| 29-04 | 1 | 0.71 |
| 06-05 | 3 | 1.75 |
| 13-05 | 10 | 9.45 |
| 21-05 | 30 | 39.1 |
| 28-05 | 50 | 112* |
| 03-06 | 100 | 134* |
| 11-06 | 200 | 402 |

*This table shows the dates of the PK results, the dose during PK sampling and the (calculated) through levels. If there was no through level sample available, through levels were extrapolated trough log-linear extrapolation (marked with *).^1^ The concentration measured on 200 mg QD might be influenced by the switch from 300 to 200 mg QD* *two days before PK measurement, as the 300 mg is expected to give higher PK levels and imatinib half-life is 18 hours, the influence of this higher dose on exposure might not have cleared. PK: Pharmacokinetic; QD: quaque die = once daily*

1. Wang Y, Chia YL, Nedelman J, Schran H, Mahon FX, Molimard M. A therapeutic drug monitoring algorithm for refining the imatinib trough level obtained at different sampling times. Ther Drug Monit. 2009;31(5):579-84.
